# Utilization of Inhaled Corticosteroids for Infants with Bronchopulmonary Dysplasia

**DOI:** 10.1371/journal.pone.0106838

**Published:** 2014-09-05

**Authors:** Jonathan L. Slaughter, Michael R. Stenger, Patricia B. Reagan, Sudarshan R. Jadcherla

**Affiliations:** 1 The Department of Pediatrics, The Ohio State University College of Medicine and Nationwide Children's Hospital, Columbus, Ohio, United States of America; 2 Center for Perinatal Research, The Research Institute at Nationwide Children's Hospital, Columbus, Ohio, United States of America; 3 The Neonatal and Infant Feeding Disorders Research Program, Nationwide Children's Hospital, Columbus, Ohio, United States of America; 4 The Department of Economics, The Ohio State University, Columbus, Ohio, United States of America; 5 Center for Human Resource Research, The Ohio State University, Columbus, Ohio, United States of America; Nottingham University, United Kingdom

## Abstract

**Objective:**

To determine demographic and clinical variables associated with inhaled corticosteroid administration and to evaluate between-hospital variation in inhaled steroid use for infants with bronchopulmonary dysplasia (BPD).

**Design:**

Retrospective Cohort Study.

**Setting:**

Neonatal units of 35 US children's hospitals; as recorded in the Pediatric Health Information System (PHIS) database.

**Patients:**

1429 infants with evolving BPD at 28 days who were born at <29 weeks gestation with birth weight <1500 grams, admitted within the first 7 postnatal days, and discharged between January 2007–June 2011.

**Results:**

Inhaled steroids were prescribed to 25% (*n* = 352) of the cohort with use steadily increasing during the first two months of hospitalization. The most frequently prescribed steroid was beclomethasone (*n* = 194, 14%), followed by budesonide (*n* = 125, 9%), and then fluticasone (*n* = 90, 6%). Birth gestation <24 weeks, birth weight 500–999 grams, and prolonged ventilation all increased the adjusted odds of ever receiving inhaled corticosteroids (p<0.05). Wide variations between hospitals in the frequency of infants ever receiving inhaled steroids (range: 0–60%) and the specific drug prescribed were noted. This variation persisted, even after controlling for observed confounders.

**Conclusions:**

Inhaled corticosteroid administration to infants with BPD is common in neonatal units within U.S. Children's hospitals. However, its utilization varies markedly between centers from no treatment at some institutions to the majority of infants with BPD being treated at others. This supports the need for further research to identify the benefits and potential risks of inhaled steroid usage in infants with BPD.

## Introduction

Inhaled corticosteroids, commonly used to prevent and reduce asthma symptoms, are sometimes prescribed to very low birth weight (VLBW) preterm infants with the hope they will be similarly useful in preventing or ameliorating bronchopulmonary dysplasia (BPD) [1]. Systemic steroids reduce BPD incidence, but prolonged high-dose regimens have been linked to neurodevelopmental delays [2]. A potential benefit of inhaled corticosteroid administration is a reduction in harmful, systemic side effects relative to parenteral or enteric formulations [3].

Nevertheless, data on the effectiveness of these drugs and their potential for adverse effects in this fragile population of preterm neonates are extremely limited. Multiple Cochrane Reviews have recommended against the routine use of inhaled corticosteroids for the prevention or treatment of BPD given a lack of supporting evidence [4]–[8].

Two provider surveys, one in Germany and another in the United States, documented that inhaled corticosteroids are prescribed frequently to preterm infants to treat or prevent BPD [9], [10]. However, prescribing patterns for inhaled steroid administration to infants with BPD, including frequency of use, average length of treatment, variables associated with administration, and inter-hospital variations in use, have never been fully reported. Pharmacoepidemiologic knowledge of current inhaled corticosteroid utilization patterns, including inter-institutional differences in their use to prevent or treat BPD, will help inform the design of patient-centered trials to examine the comparative effectiveness of inhaled corticosteroids administration for improving BPD outcomes.

Therefore, our investigational objectives were to (1) determine demographic and clinical variables associated with inhaled corticosteroid administration, and to also (2) determine between-hospital variation in inhaled corticosteroid use for infants with bronchopulmonary dysplasia (BPD) including hospital-specific treatment frequency and inpatient treatment duration.

## Methods

### Study Design

We performed a retrospective cohort study to evaluate inhaled steroid use in infants with BPD. Infants were considered to have evolving BPD if they survived until at least 28 days of age and received respiratory support for the first 28 consecutive days of life via mechanical ventilation, CPAP, and/or supplemental oxygen [11]. The cohort, described previously by Slaughter *et al* [11], included infants with evolving BPD born prior to 29 weeks of gestation with a birth weight <1500 grams and admitted to neonatal intensive care units (NICUs) in children's hospitals at ≤7 days of age with discharge dates from January 2007–June 2011, as recorded in the Children's Hospital Association (CHA) Pediatric Health Information System (PHIS) database (Shawnee Mission, Kansas). We chose our gestational age (<29 weeks) and birth weight (<1500 grams) cut-offs to include >97% of infants with BPD [12]. We excluded infants admitted after seven days of age to minimize exposure to unmeasured inhaled steroid treatment at outside hospitals and only included those who lived ≥28 days, the earliest age at which BPD may be assigned [13]. Since an infant's respiratory condition at BPD onset was more likely to influence a clinician's decision regarding inhaled steroid usage throughout the remainder of their hospitalization than a 36 week outcome measurement, we chose to define BPD at its 28 day onset, prior to severity staging at 36 weeks corrected age. For reference, we also determined the frequency of mild, moderate, and severe BPD at 36 weeks.

### Data Source

The PHIS database contains administrative, billing, and record-review data including patient demographics, diagnoses, procedures, and medications labeled by route of administration from 43 freestanding U.S. Children's Hospitals, which account for 85% of all national freestanding children's hospitals (Children's Hospital Association, Shawnee Mission, KS).

The study sample for patient-level analyses consisted of 1429 admissions from 35 hospitals with cases of evolving BPD at 28 days, as defined above. To prevent bias from the overweighting of institutions with smaller BPD populations, between-hospital comparisons based on the mean proportion of hospital inhaled steroid use were confined to the 15 hospitals with at least 25 BPD cases. These 15 institutions represented 86% (*n* = 1226) of the study-sample.

### Study Variables

Daily drug-specific inhaled steroid administration, mechanical ventilation, CPAP, oxygen use, as well as length of stay and demographic variables were determined from each hospital's daily charge records as included in PHIS. Thompson-Reuters Healthcare (Ann Arbor, Michigan), the PHIS data processing partner, maps each hospital's daily charge codes to a common classification system, the Clinical Transaction Classification (CTC) Codes to ensure comparability of charge-level data between institutions. CTC codes evaluated included: inhaled beclomethasone (154013.42), inhaled budesonide (154021.42), inhaled fluticasone (154055.42), inhaled triamcinolone (154087.42), inhaled mometasone (154073.42), mechanical ventilation (521166), CPAP (521162), oxygen delivery by cannula, tent, or mask (521171). We evaluated the use of all inhaled steroids included in the PHIS database within our cohort population and excluded those with <1% frequency of use (triamcinolone, mometasone) from further analysis. Review of routes and dosages indicated that, with the exception of one hospital, patients received budesonide via nebulized solution, while beclomethasone and fluticasone were administered via metered dose inhaler (MDI).

International Statistical Classification of Diseases, Ninth Revision (ICD-9) Codes were used to determine the diagnoses of patent ductus arteriosus (PDA) (747.0), intraventricular hemorrhage (IVH) (772.1), and necrotizing enterocolitis (NEC) (777.5). Weeks of gestation were defined by ICD-9 codes: <24 (765.21); 24 (765.22); 25–28 (765.23); 27–28 (765.25) as were the four categorical variables for birth weight (grams), <500, 500–749, 750–999, 1000–1499. If ICD-9 codes were not reported or were contradictory, information from the patient profile was used. Gender was defined by patient profile.

### Statistical Analysis

All analyses were conducted using Stata 12.1(StataCorp, College Station, Texas). Logistic regression was used to determine unadjusted odds ratios for bivariate associations between inhaled steroid use and neonatal demographic/clinical risk factors. Multivariable mixed logistic regression modeling with a random intercept for each hospital was used to adjust odds ratios for confounding variables and to evaluate the contribution of within-hospital clustering to variation in inhaled steroid administration. The model was created by purposeful selection and included gestational age, gender, duration of mechanical ventilation or CPAP, IVH, PDA, and NEC as variables. A second model was created with birth weight replacing gestational age to obtain adjusted odds ratios (aORs) for birth weight. This was done to avoid multicollinearity between birth weight and gestational age. All statistical testing was two-sided and an alpha-level of 0.05 was considered significant. We report the median measure of central tendency because the distributions for all inhaled steroid formulations are skewed to the right, indicating wide variation in treatment duration.

### Ethics Review

The Nationwide Children's Hospital Institutional Review Board determined that this was not human subjects' research, because it was an analysis of a pre-existing, de-identified dataset and involved no patient contact.

## Results

A total of 1429 infants met the criteria for BPD at 28 days, of which 352 (25%) were treated at least once with inhaled steroids. Inhaled beclomethasone was administered to 14% (194) infants, budesonide to 9% (125), and fluticasone to 6% (90). In the unadjusted analysis, birth weight <1000 grams, decreasing gestation, increasing exposure to CPAP or mechanical ventilation, PDA, and NEC were associated with significantly increased odds of inhaled steroid treatment. Gender, race, study year, and a history of intraventricular hemorrhage were also examined, but were not statistically significant. When all variables were included in a multivariable logistic regression model with a random intercept to adjust for confounding and within-hospital clustering (Table 1), the odds of an infant with BPD ever receiving inhaled steroids were significantly associated with birth at ≤24-weeks gestation, birth weight between 500–999 grams, and increasing duration of CPAP or mechanical ventilation. There was no significant association between inhaled steroid administration and birth weight <500-grams. This could be related to a small sample (*n* = 40) and increased inpatient mortality risk (risk ratio: 2.93; P = 0.004) relative to other infants in the cohort.

**Table 1:** Multivariable Adjusted Odds of Receiving Inhaled Steroids.

Variable	Ever Received Inhaled Steroids (Adjusted Odds Ratio, 95% CI)
**Gestation**	
27–28 weeks (Reference)	-
25–26 weeks	1.45 (1–2.1)
≤24 weeks	2.25 (1.43–3.54)*
**Birth Weight (grams)**	
1000–1499 gms (Reference)	-
750–999 gms	1.96 (1.25–3.08)*
500–749 gms	3.16 (1.92–5.2)*
<500 gms	2.05 (0.81–5.18)
**Gender**	
Male (Reference)	-
Female	0.84 (0.61–1.15)
**Days on CPAP or Mechanical Ventilation**	
≤20 days (Reference)	-
21–35 days	2.26 (1.3–3.93)*
36–53 days	5.63 (3.18–9.97)*
≥54 days	15.81 (8.44–29.59)*
**Major Comorbidities**	
IVH	0.94 (0.68–1.29)
NEC	0.91 (0.62–1.33)
PDA	1.14 (0.82–1.6)

Odds ratios (95% C.I.) except those for *Major Comorbidities* are reported relative to the reference group indicated by (−). Adjusted odds ratios (aORs) were determined using a mixed-effects logistic regression model with a random intercept for hospital, in which all variables in the table were fit in the model except for gestational age. Gestational age aORs were determined by a second model, which included all variables except for birth weight, in order to avoid multicollinearity between birth weight and gestational age.

*Statistically significant at alpha  = 0.05.

### Cohort at 36 Weeks Postmenstrual Age

At 36 weeks postmenstrual age, 96% (*N* = 1376) of the cohort survived with 313 discharges prior to 36 weeks. Oxygen requirement (moderate BPD as defined by Ehrenkranz *et al* [14]) was noted in 34% (473) and 22% (316) required CPAP or mechanical ventilation (severe BPD [14]). Inhaled steroids were administered to 12 (23%) of the 53 infants who died prior to 36 weeks. Of surviving infants, 43 (7%) with mild BPD (off oxygen or discharge at <36 weeks; *N* = 587), 68 (14%) with moderate BPD, and 47 (15%) with severe BPD received inhaled steroids at least once. Death occurred in 24 hospitalized infants after 36 weeks.

### Between-Hospital Variation In Inhaled Steroid Treatment

**Figure 1:**
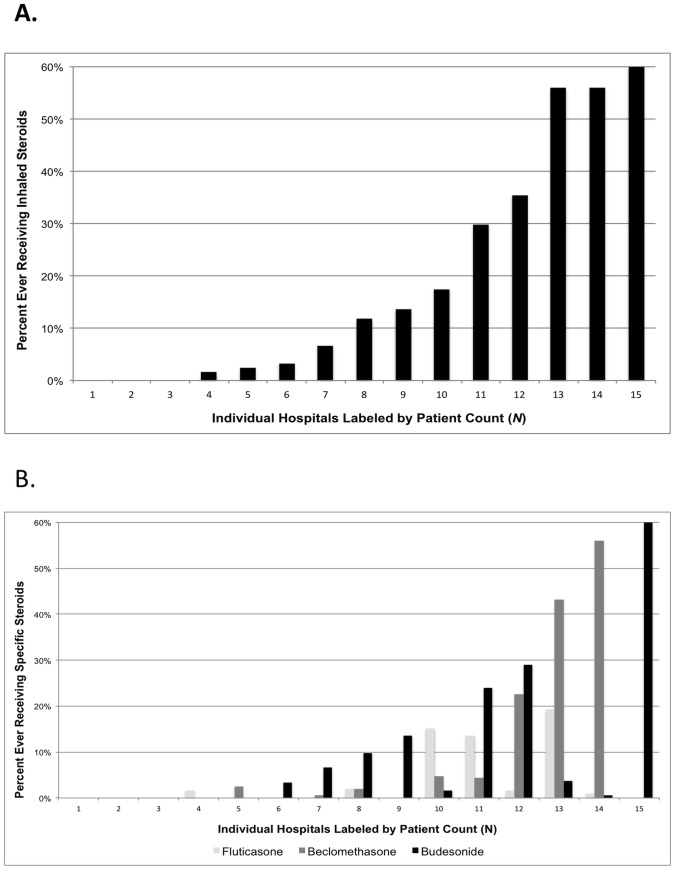
Percentage of infants that ever received inhaled steroids (nebulized or MDI) during their hospital stay, by hospital. Range: 0 to 60% (median: 12%). Hospitals were included if at least 25 patients developed BPD during the study period (*N* = 15).

Inhaled Steroid use for infants with BPD varied between children's hospitals. By hospital (*N* = 15), the percentage of patients ever receiving these medications ranged from 0–60% (median: 12; IQR: 2%–35%) (*Figure 1a*) with 3 hospitals never prescribing them. Variation in inhaled steroid course use between hospitals persisted even after controlling for length of CPAP/mechanical ventilation exposure, birth weight, gestational age, IVH, PDA, and NEC in our multivariable logistic regression models with random intercepts (Table 1). The intraclass correlation coefficient (ICC), a measure of the proportion of total variance in inhaled steroid use due to variation between hospitals, indicated that clustering by hospital was a significant component of the overall variation in inhaled steroid utilization [ICC = 0.56 (95% CI: 0.38–0.72)]. In addition, the specific inhaled steroid prescribed varied by hospital (*Figure 1b*).

### Timing of Inhaled Corticosteroid Initiation

**Figure 2:**
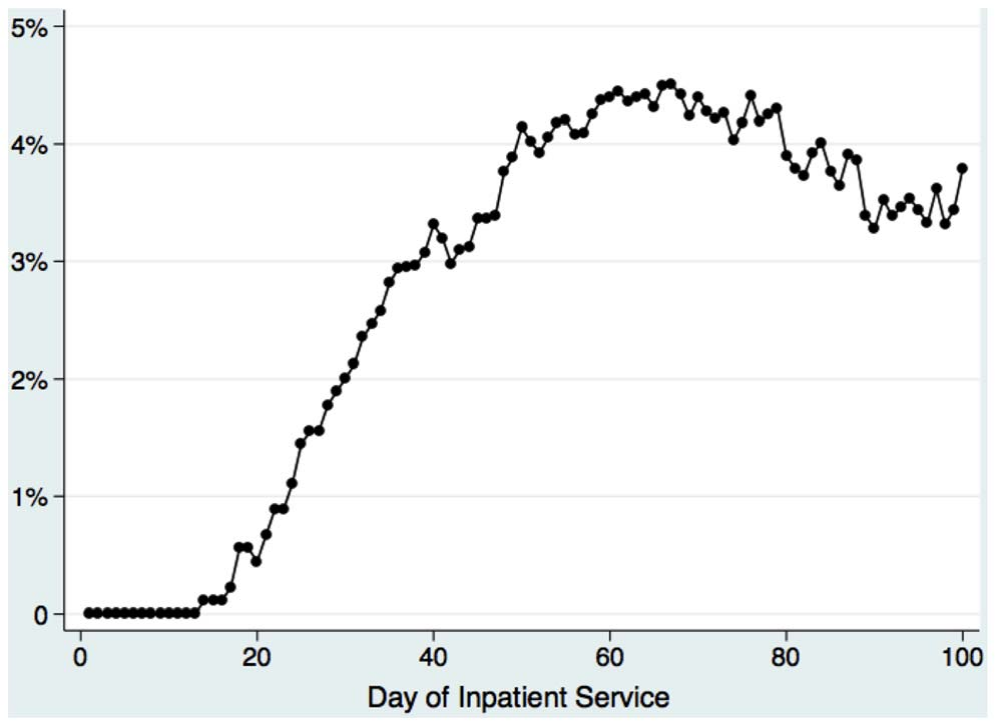
Percentage of infants with evolving BPD having ever received inhaled corticosteroids, by day of hospitalization.

*Figure 2* demonstrates the percentage of infants with BPD having ever received inhaled steroids by hospital day. Use sequentially increased from the third week of hospitalization until ultimately peaking on day 67, when 4.5% had been treated.

### Duration of Nebulized Budesonide Therapy

Since MDI treatments are billed per inhaler device, we were only able to determine cumulative days for nebulized budesonide treatments, which are billed per administration. Treated patients received a median of 35 (25^th^–75^th^ percentiles: 10–57) (range 1–507) days of nebulized budesonide prior to discharge. Those ever treated with MDIs, received a median of 1 inhaler (range 1–2) prior to hospital discharge.

## Discussion

Our investigation indicates that despite a lack of supporting evidence, inhaled corticosteroids are commonly administered to infants with BPD at US children's hospitals. Furthermore, prescribing patterns vary markedly between institutions. Other key findings were: 1) Overall, 25% of the cohort received an inhaled corticosteroid, with increasing usage throughout the first two months of hospitalization. 2) We found that duration of mechanical ventilation or CPAP exposure, a marker of respiratory disease severity, was the greatest predictor of inhaled corticosteroid exposure.

The proportion of infants with BPD receiving inhaled corticosteroids varied widely between hospitals, ranging from 0% (at three hospitals) to 60%. This variation persisted even after controlling for measured confounders. Beclomethasone was used at 7 hospitals, and was the most frequently administered inhaled corticosteroid. Budesonide was used most commonly at 4 hospitals and fluticasone at 3 hospitals.

Proposed mechanisms of beneficial steroid effects for BPD include reduction of pulmonary inflammation with subsequently reduced edema and fibrosis, enhancement of surfactant and antioxidant enzyme production, decreased bronchospasm, and increased vitamin A levels [1]. Systemic corticosteroid administration reduces BPD incidence, but prolonged, higher dose therapy is linked to neurodevelopment delay. Inhaled steroids improve asthma symptoms by reducing pulmonary inflammation without the severe side effects of systemic steroids. However, published trial data have not shown a reduction of BPD following inhaled steroid administration [5]–[8]. These trials vary considerably in patient enrolment criteria, reported outcomes, medication, dose, treatment duration, and mode of drug delivery. Some studies demonstrated improvement in various physiologic measures of respiration including improved mean peak inspiratory pressure in ventilated infants randomized to budesonide [15], improved pulmonary resistance in ventilated preterm infants receiving nebulized beclomethasone [16] or inhaled dexamethasone [17], and improved lower chest radiograph scores following inhaled fluticasone [18]. In one trial, early beclomethasone treatment was associated with lower subsequent systemic glucocorticoid utilization [19]. Cochrane meta-analyses concluded that the use of inhaled corticosteroids for premature infants with or at risk for BPD cannot be recommended after finding no significant improvement in survival, composite mortality or BPD outcome, or total days on mechanical ventilation or supplemental oxygen following treatment [5]–[8].

Limitations of neonatal inhaled glucocorticoid studies include uncertain aerosol delivery and variable lung deposition. Particle size, delivery device, and administration site along the ventilator or CPAP [20] circuit affect drug delivery and deposition [21]. The average pulmonary deposition or bioavailability for nebulized medications to both intubated and spontaneously breathing infants has been estimated to be less than 1% [22], [23]. The Neonatal EURopean Study of Inhaled Steroids (NEuroSIS) study, an on-going trial and largest to date, aims to maximize budesonide delivery to infants on CPAP and mechanical ventilation with a spacer device, in order to evaluate its impact on BPD development and 18–22 month neurodevelopmental outcome [24]. In addition to delivery concerns, inhaled glucocorticoid effectiveness varies by patient. Roughly 30% of asthma patients exhibit some degree of insensitivity to inhaled steroids, thought to be due to effect modification of steroid clearance by polymorphisms in the Cytochrome P450, family 3, subfamily A, (CYP3A) enzymes responsible for steroid metabolism [25]–[27].

Compared to systemic corticosteroids, inhaled glucocorticoids have fewer adverse effects. Nonetheless, there is some systemic absorption from the lungs and, to a lesser extent, the gastrointestinal tract [28]. Corticosteroids are also potent inhibitors of linear growth and studies have shown evidence for pituitary-adrenal suppression in premature infants receiving inhaled steroids [29], [30]. Kelly et al found that the use of inhaled glucocorticoids in prepubertal children led to decreased growth velocity and adult height reduction [31]. Inhaled corticosteroid use has been associated with small decreases in bone mineral accretion in boys [32] and higher systolic blood pressure was reported for premature infants after 28 days of treatment with inhaled fluticasone compared to placebo [18]. Their impact on the growth of developing preterm infants is unknown. Inhaled steroids may also increase the risk of infection, which is worrisome given the relative immunocompromised state of preterm infants. Adults receiving inhaled corticosteroids have an increased pneumonia risk [33]. A recent case report described a term infant who developed Candida pneumonia after being treated with inhaled steroids [34] and a case series described inhaled steroid-associated tongue hypertrophy [35]. Future pharmacoepidemiology studies are needed to assess side effects [36], [37].

In this retrospective, observational investigation, we were unable to establish the causal effects of inhaled corticosteroids on important clinical outcomes such as duration of ventilation and oxygen use, severity of BPD, and length of stay. All of these outcomes were associated with increased inhaled corticosteroid use in our cohort, but our ability to control for selection bias due to severity of respiratory illness was limited to observed covariates. We did not observe any adverse effects of inhaled corticosteroid use within the cohort, but our ability to evaluate for side effects was limited by the outcomes recorded in PHIS. Clinical data for detecting and measuring reported adverse effects of corticosteroids, such as weight loss, linear growth measurements, pituitary hormones and other laboratory results, and radiography including bone density measurements are not currently available within PHIS. Our diagnoses, including BPD, were based on hospital records. Potential recording errors may potentially reduce the diagnostic accuracy. Although we used daily, hospital charge data to determine days receiving oxygen, mechanical ventilation, CPAP, and dates of inhaled corticosteroid administration, we relied on less specific ICD-9 codes for IVH, PDA, and NEC diagnoses. Even though PHIS data are rigorously screened for errors and rejected if quality thresholds are not met, they were initially collected for administrative purposes instead of specifically for research.

Despite these limitations, our cohort study has multiple strengths. It benefits from a large sample size and nationally representative sample of children's hospitals included in the PHIS database, as well as measures taken by CHA to ensure data quality. Our findings are likely generalizable to most NICUs within large US children's hospitals and these baseline findings will serve to inform the design of prospective, comparative effectiveness investigations to determine whether long-term use of inhaled corticosteroids in BPD patients is beneficial.

## Conclusions

Inhaled corticosteroid administration to infants with BPD is common at US Children's hospitals. The frequency of inhaled corticosteroid administration varies markedly by institution even after adjustment for confounding variables. While a multicenter, international, double-blinded randomized controlled trial evaluating the efficacy and safety of inhaled budesonide in infants 23–27 weeks gestation who require positive pressure ventilation is underway [24], published trial data do not support inhaled steroid routine use to prevent or treat infants with BPD [2]. Further research is needed to determine the effectiveness and safety of long-term inhaled corticosteroid therapy for BPD prevention and treatment, and enable evidence-based recommendations.
